# Congenital mixed hiatal hernia: A case report of an atypical cause of neonatal vomiting

**DOI:** 10.1002/jpr3.12042

**Published:** 2024-01-25

**Authors:** Katia N. Estrada‐Medrano, Sergio J. Fernández‐Ortiz, Oscar Tamez‐Rivera, Enrique G. Villarreal

**Affiliations:** ^1^ Department of Pediatrics Secretaría de Salud del Gobierno del Estado de Nuevo León, Hospital Regional de Alta Especialidad Materno Infantil Monterrey Nuevo León México; ^2^ Tecnologico de Monterrey, Escuela de Medicina y Ciencias de la Salud Monterrey Nuevo Leon Mexico

**Keywords:** congenital diaphragmatic defect, diaphragmatic hernia, hiatal hernia, infant

## Abstract

Congenital mixed hiatal hernia is a disorder that combines features of both sliding and paraoesophageal hernias. The precise incidence of congenital mixed hiatal hernia during the pediatric and neonatal period remains uncertain, making diagnosis challenging within this age cohort. This case presents a 15‐day‐old female with an 8% postnatal weight loss and apost‐feeding vomiting. An upper gastrointestinal series, computer tomography, and upper endoscopy revealed a mixed hiatal hernia. The patient underwent a laparoscopic herniorrhaphy and Nissen fundoplication achieving successful resumption of complete oral feeding before discharge. Diagnosis and management of this condition in neonates remain challenging due to its rarity and variable clinical presentations. This report emphasizes the importance of early recognition, accurate diagnosis, and tailored management strategies in the neonatal period. Further research, with a collaborative effort between pediatricians and surgeons, is needed to refine diagnostic criteria, establish evidence‐based management approaches, and improve outcomes for affected children.

## 1 INTRODUCTION

Congenital mixed hiatal hernia is a disorder that combines features of both sliding and paraoesophageal hernias.^1^ In this type of hiatal hernia, not only does a portion of the stomach slide upward through the esophageal hiatus (sliding hernia), but there is also an adjacent pouch of the stomach that herniates through the diaphragm alongside the esophagus (paraoesophageal hernia). The mixed hiatal hernia differs in terms of anatomy, epidemiological patterns, symptomatology, complications, and management.^2^ The precise incidence of congenital mixed hiatal hernia during the pediatric and neonatal period remains uncertain, making diagnosis challenging within this age cohort.

This case is considered intriguing due to its uncommon and nonspecific clinical presentation in this age cohort. This case can shed light on how to approach the diagnostic and therapeutic management of a condition that may manifest solely as vomiting in the neonatal age.

### 1.1 Case report

A 15‐day‐old female was admitted to the emergency room due to irritability and oral intolerance, which progressed to postfeeding nonbloody and nonbilious vomiting. After an ineffective response to the medical interventions prescribed during a prior consultation, which included a transition to an extensive hydrolyzed formula and a proton pump inhibitor treatment regimen, the patient's mother sought additional assistance, which led to our medical service. Following a comprehensive physical examination, a significant observation was an 8% postnatal weight loss. The weight decreased from 3260 g at birth (42nd percentile) to 2970 g at the time of evaluation (11th percentile). Abdominal examination revealed no abnormalities. In view of this clinical picture, the decision was made to admit the patient for a comprehensive diagnostic assessment.

**Figure 1:**
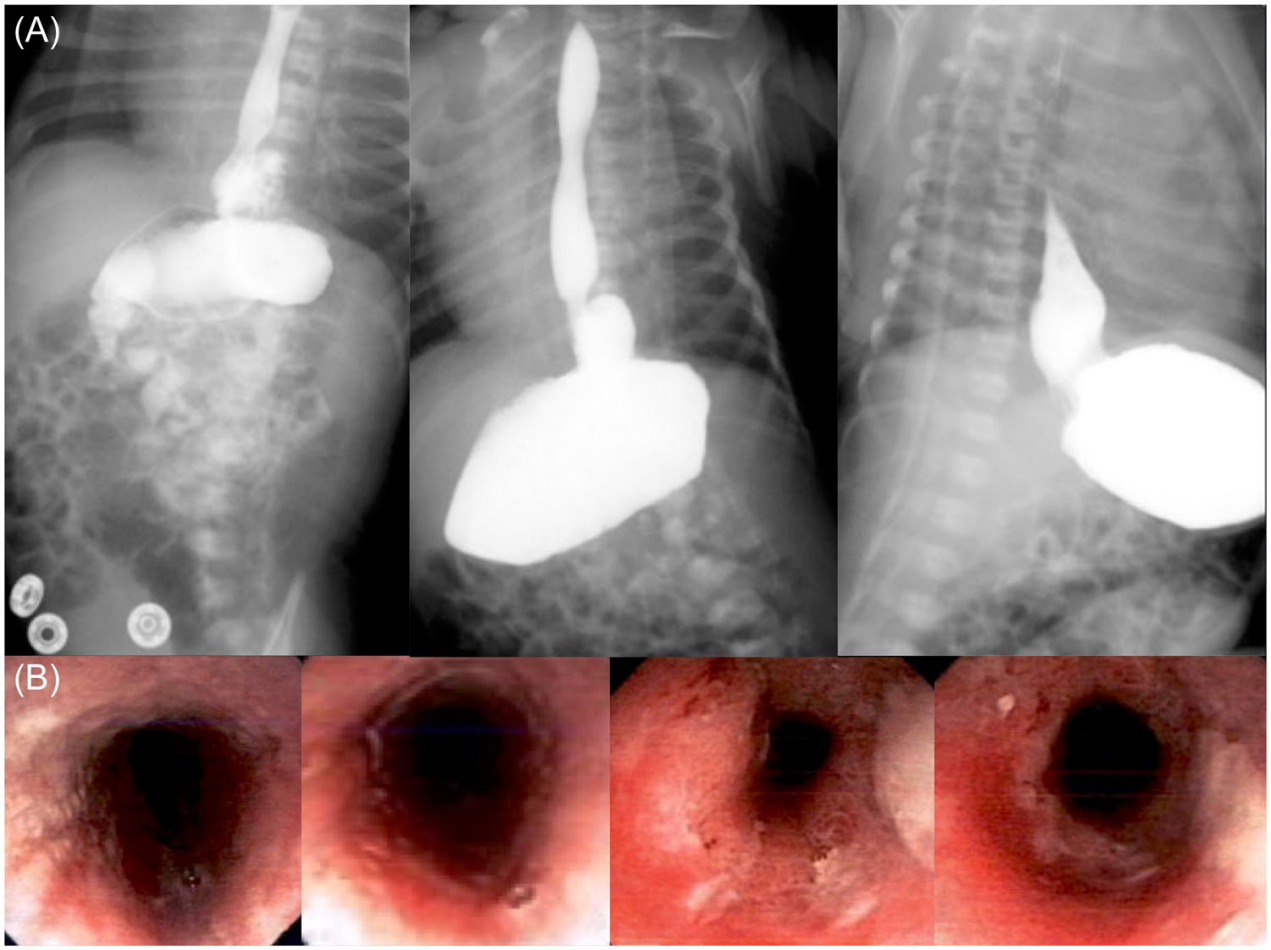
Diagnostic work‐up performed in the case report. (A) Upper gastrointestinal series illustrates a mixed hiatal hernia with reflux extending to the upper third segment of the esophagus, without evidence of aspiration. (B) Upper endoscopy displays an ulcerated esophagitis resulting from a mixed hiatal hernia.

An upper gastrointestinal series was conducted, revealing an image consistent with mixed hiatal hernia, illustrating the protrusion of the stomach fundus through the esophageal hiatus into the thoracic cavity (Figure 1A). Seeking further anatomical clarification, an abdominal computed tomography (CT) scan was performed, confirming the presence of the gastroesophageal junction above the diaphragm, along with the gastric fundus. Subsequently, an upper endoscopy revealed mucosal damage resulting from a mixed hiatal hernia (Figure 1B). The findings disclosed an ulcerated esophagitis, further underlining the clinical significance of the hernia.

To address the anatomical anomaly and mitigate the gastroesophageal reflux, the patient underwent a laparoscopic herniorrhaphy and Nissen fundoplication. These interventions included the reduction of the herniated content and resection of the esophageal sac. The patient presented a favorable postoperative course without complications, achieving successful resumption of complete oral feeding without vomiting before discharge. One‐month postsurgery, the patient exhibited a weight gain of 1800 g, with no reported history of reflux.

## 2 DISCUSSION

Hiatal hernia is defined as the protrusion of abdominal cavity contents into the mediastinum through a widening of the right crus of the diaphragm.^3^ The historical context of this condition traces back to 1926 when Åke Åkerlund introduced the term *hiatus hernia* and proposed an initial classification system.^3^ This original classification included three subtypes (in parenthesis, the textual definition of the original article): (I) sliding hernia (*congenitally shortened esophagus*), (II) paraesophageal hernia (*esophagus not too short, but passes beside the hernia without being part of it*), and (III) other hiatal hernia (*esophagus not too short, with the portion of the gullet forming part of the hernia*). Decades later, the third subtype was renamed as mixed hiatal hernia. Advancements in imaging technologies and endoscopic techniques have contributed to the evolution of this classification.

**Figure 2:**
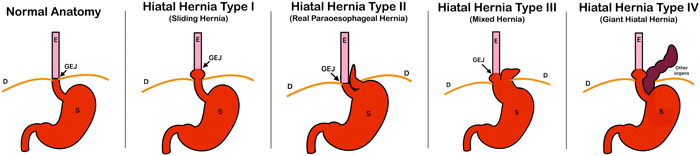
Classification of hiatal hernia. D, diaphragm; E, esophagus; GEJ, gastroesophageal junction; S, stomach.

The current medical literature embraces a more nuanced classification, outlining four distinct subtypes (Figure 2). This updated classification accounts for variations in anatomy, incidence, clinical presentation, and management strategies (Table 1). The patient described in this case report was diagnosed with a mixed hiatal hernia due to the presence of features of both types I and II hiatal hernias. This case underscores the complexity of hiatal hernia presentation and highlights the value of a comprehensive classification system in guiding accurate diagnosis and tailored management.^1^

**Table 1:** Current anatomic classification of hiatal hernias with description of anatomical features, epidemiological distribution, and treatment strategies.

	Subtype	Also known as	Anatomical features	Distribution in Adults/Children	Treatment
Sliding hernia	Type I	Sliding hernia	Characterized by a widening of the muscular hiatal tunnel and laxity of the phrenoesophageal membrane. This anatomical configuration permits an upward herniation of a section of the cardia.	95%/Unknown	Usually asymptomatic and, therefore, medical treatment for gastroesophageal reflux might be enough. If symptoms persist, fundoplication surgery is indicated.
Paraoesophageal hernia	Type II	Real paraoesophageal hernia	Localized defect in the phrenoesophageal membrane, this condition retains the gastroesophageal junction anchored to the preaortic fascia and the median arcuate ligament.	0.4%/Unknown	Asymptomatic: The management of asymptomatic or minimally symptomatic patients is a subject of debate. Expert consensus suggests that elective surgery should be considered based on factors like age and comorbidities, favoring those that are previously healthy and young. Symptomatic: For symptomatic patients, particularly those with obstructive symptoms or volvulus, prompt surgical repair is recommended. Symptomatic individuals face an elevated risk of complications, including perforation.
	Type III	Mixed hernia	This type exhibits features of both type I and type II hernias. As the hernia expands progressively through the hiatus, the phrenoesophageal membrane stretches. This results in the displacement of the gastroesophageal junction above the diaphragm, causing the sliding component of this hernia.	4.5%/Unknown	
	Type IV	Giant hiatal hernia	Characterized by a substantial defect in the phrenoesophageal membrane, this type permits the inclusion of other organs within the hernia sac. These organs may encompass the colon, spleen, pancreas, and small intestine.	0.1%/Unknown	

In contrast with adults, the precise incidence within the pediatric population remains unknown. Only a handful of studies have delved into this, with the most extensive study comprising a cohort of 59 children.^2^, ^4^, ^5^, ^6^ Intriguing insights emerged from these investigations: a lack of gender predilection; early diagnosis; noteworthy associations with other anatomical malformations; and a correlation was observed with genetic conditions, such as Marfan syndrome and trisomies. Medical literature also includes case reports documenting familial hiatal hernias spanning a multiple generations within the same family.^7^

Clinical presentation can vary significantly.^8^ Some cases may be asymptomatic, incidentally discovered during imaging tests. Conversely, it can lead to symptoms attributed to gastroesophageal reflux disease. However, in the context of paraesophageal/mixed hernias, a distinct set of severe symptoms often emerges.^9^ These include recurrent respiratory infections, bronchoaspiration, bronchial obstruction, vomiting, melena, failure to thrive, dysphagia, and early satiation. Among the most frequent complications observed are gastric volvulus, pyloric obstruction, and gastric strangulation. While rare, these complications can extend to necrosis and perforation. Other cases documented in the literature have shown that neonates with hiatal hernia might present solely with vomiting and weight loss, such as our case.^10^ This underscores the significance of considering this condition in the differential diagnosis whenever neonates exhibit persistent vomiting.

Due to its rarity in the pediatric population, guidelines for this age group have yet to be established. As of now, the diagnostic approach and management of hiatal hernia in children rely on insights from adult literature. The Society of American Gastrointestinal and Endoscopic Surgeons formulated a guideline that offer recommendations based on a comprehensive evaluation rethrough a systematic review.^8^ Experts emphasize the judicious use of diagnostic modalities that will impact clinical management. While intraoperative visualization remains the gold standard, it is rarely performed solely for diagnostic purposes. Instead, noninvasive and accurate diagnostic methods are employed prior surgery. These modalities include contrast esophagogram, upper gastrointestinal series, esophagogastroduodenoscopy, CT, and esophageal manometry. Treatment recommendations are based upon the type of hernia and clinical presentation (detailed in Table 1).

Congenital mixed hiatal hernia is a complex clinical scenario. Diagnosis and management of this condition in neonates remain intricate endeavors due to its rarity and variable clinical presentations. This report emphasizes the importance of early recognition, accurate diagnosis, and tailored management strategies in this unique pediatric population. Further research, with a collaborative effort between pediatricians and surgeons, is needed to refine diagnostic criteria, establish evidence‐based management approaches, and ultimately improve outcomes for affected children.

## CONFLICT OF INTEREST STATEMENT

The authors declare no conflict of interest.

## ETHICS STATEMENT

Written consent was obtained and signed for the publication of this case report. Informed Consent for publication of the details of this case was provided by the patient's mother (legal guardian).
